# Early experiences of integrating an artificial intelligence-based diagnostic decision support system into radiology settings: a qualitative study

**DOI:** 10.1093/jamia/ocad191

**Published:** 2023-09-25

**Authors:** Nuša Farič, Sue Hinder, Robin Williams, Rishi Ramaesh, Miguel O Bernabeu, Edwin van Beek, Kathrin Cresswell

**Affiliations:** Usher Institute, University of Edinburgh, Edinburgh, United Kingdom; Usher Institute, University of Edinburgh, Edinburgh, United Kingdom; Institute for the Study of Science, Technology and Innovation, University of Edinburgh, Edinburgh, United Kingdom; Department of Radiology, Royal Infirmary Hospital, Edinburgh, United Kingdom; Usher Institute, University of Edinburgh, Edinburgh, United Kingdom; The Bayes Centre, University of Edinburgh, Edinburgh, United Kingdom; Centre for Cardiovascular Science, Edinburgh Imaging and Neuroscience, University of Edinburgh, Edinburgh, United Kingdom; Usher Institute, University of Edinburgh, Edinburgh, United Kingdom

**Keywords:** artificial intelligence, radiology, clinical decision support, diagnostic

## Abstract

**Objectives:**

Artificial intelligence (AI)-based clinical decision support systems to aid diagnosis are increasingly being developed and implemented but with limited understanding of how such systems integrate with existing clinical work and organizational practices. We explored the early experiences of stakeholders using an AI-based imaging software tool Veye Lung Nodules (VLN) aiding the detection, classification, and measurement of pulmonary nodules in computed tomography scans of the chest.

**Materials and methods:**

We performed semistructured interviews and observations across early adopter deployment sites with clinicians, strategic decision-makers, suppliers, patients with long-term chest conditions, and academics with expertise in the use of diagnostic AI in radiology settings. We coded the data using the Technology, People, Organizations, and Macroenvironmental factors framework.

**Results:**

We conducted 39 interviews. Clinicians reported VLN to be easy to use with little disruption to the workflow. There were differences in patterns of use between experts and novice users with experts critically evaluating system recommendations and actively compensating for system limitations to achieve more reliable performance. Patients also viewed the tool positively. There were contextual variations in tool performance and use between different hospital sites and different use cases. Implementation challenges included integration with existing information systems, data protection, and perceived issues surrounding wider and sustained adoption, including procurement costs.

**Discussion:**

Tool performance was variable, affected by integration into workflows and divisions of labor and knowledge, as well as technical configuration and infrastructure.

**Conclusion:**

The socio-organizational factors affecting performance of diagnostic AI are under-researched and require attention and further research.

## Introduction

Artificial intelligence (AI) in healthcare involves applying machine learning techniques to identify and uncover patterns in multidimensional data to improve health outcomes and patient experience.^1^ Empirical evidence suggests promise in helping improve early disease and adverse event detection.^2^ However, evidence on outcomes is mixed as efforts to develop and launch new tools across different settings have not been matched by studies of their impact on care delivery and patient outcomes.^3–6^

Computerized clinical decision support systems (CDSSs) are software applications that provide healthcare professionals with real-time, evidence-based information and recommendations to aid in clinical decision-making.^7^ CDSSs can be based on explicitly defined rules and medical knowledge or based on machine learning, leveraging advanced computational techniques to learn patterns from data, and make decisions based on statistical inference.^8^ CDSSs have shown significant potential in improving practitioner performance and patient outcomes,^9^ but they have also been associated with issues surrounding alert-fatigue and adverse impacts on work practices (eg, when hard stops cannot be overridden).^10^^,^^11^ CDSS can be knowledge based or data driven. Knowledge-based CDSS relies on predefined rules, guidelines, and medical knowledge to provide recommendations or alerts to healthcare professionals. Data-driven CDSS uses machine learning algorithms to analyze large volumes of data and learn patterns to provide recommendations and predictions.

AI applications in radiology, including chest computed tomography (CT) scans and mammography, are receiving increasing attention.^12–17^ AI in radiology is now the top specialty of approved medical AI applications according to the United States (US) Food and Drug Administration (FDA) with 70.3% of all AI devices developed in this area.^18^ Radiology requires diagnosis and decision-making under uncertainty and AI may help automate some of the labor-intensive tasks such as radiograph interpretation and reporting.^19^^,^^20^ AI algorithms applied to chest radiographs have, for example, been shown to aid diagnosis and improve clinician performance.^21–24^ However, the processes involved in integrating AI-based imaging systems within existing professional workflows and patient care pathways are still unclear.^8^^,^^25^^,^^26^

Existing qualitative research focusing on diagnostic decision support reveals tensions around different types of users and use cases.^27–29^ For example, radiologists and radiographers hold different views about the prospects of AI in their practice. A recent study has shown that radiologists were better informed about the emerging AI in their field than radiographers and had more positive attitudes toward the technology, whereas radiographers were concerned that AI might jeopardize their roles in the future.^27^ Similarly, a large international survey about AI technology with over 1000 radiologists and radiology residents revealed that clinicians with limited experience with AI associated it with fear, in contrast to intermediate or advanced AI users whose attitudes toward the technology were positive, including holding a belief that AI skills should be part of radiology training.^28^ Studies with patients highlight their limited knowledge surrounding the use of AI in radiology and in their healthcare.^29^

There are currently no studies on how AI used for lung imaging is implemented, adopted, and integrated into existing workflows.^30^ Work in other areas highlights potential issues, such as low specificity resulting in a high volume of false positives and consequent requests for additional investigations (creating anxiety and in some cases resulting in unnecessary invasive procedures, such as biopsies).^31^

We therefore aimed to explore early experiences of implementing and using an AI-based diagnostic decision support system in chest radiology settings from a variety of stakeholder perspectives, to understand how the technology was integrated within real-world socio-organizational contexts.

We studied an AI-based diagnostic decision support tool, Veye Lung Nodules (VLN) (Aidence/RadNet), which was implemented in United Kingdom (UK) hospital-based chest radiology settings (Box 1). The tool is considered AI-based because it utilizes machine learning algorithms. VLN is currently used in 40 National Health Service (NHS) hospitals to support lung cancer screening. VLN runs in the background of the Picture Archiving and Communications System (PACS), automatically processing all eligible studies, including the most recent prior, if available. Its results are delivered directly to the PACS, as part of the original diagnostic series without the need for additional clicks. The results are available to anyone with PACS access, on or off-site.

Box 1: Description of the tool

Veye Lung Nodules (VLN) is an integrated machine-learning-based imaging software tool that aids in the detection, classification, and (size and volume) measurement of pulmonary nodules in CT scans of the chest. VLN is a Communauté Européenne (CE) certified second or concurrent reader, suitable for routine clinical practice and lung cancer screening and fully compliant with the new EU Medical Device Regulation (classified as a class IIb device). Results are automatically available during reporting for all eligible scans. It is currently used in NHS and EU centers to support lung cancer screening. Use cases include the detection and monitoring of pulmonary nodules in lung cancer screening (where the primary aim is to identify lung nodules in an at-risk population) and routine hospital practice (where nodule detection is often incidental to the primary purpose for which the CT chest scan was ordered). In the former, the scan is conducted for patients previously identified with nodules, whilst in the latter, the scan is conducted for other chest conditions (eg, heart, trauma) and patients are asymptomatic for lung cancer but may have multiple other symptoms. The integration with workflows is the same across use cases. Results are transmitted to the PACS and can be seen when examining the scan on the reader. The target user group includes radiologists and radiographers with varying levels of expertise.

## Methods

Our work was part of a mixed-methods study exploring the implementation of VLN in multiple hospitals. Aidence (the developer) was awarded funds under the NHS AI Award program in 2020 to undertake a real-world evaluation of its software to generate evidence supporting a full health technology appraisal by the National Institute for Health and Care Excellence (NICE). This qualitative study was part of this evaluation. Other aspects included an assessment of clinical impact and health economic modeling.

We conducted a qualitative semistructured interview study with clinicians who used the software, organizational implementers, strategic decision-makers, suppliers, patients with long-term chest conditions, and experts in the field, to obtain a holistic view on how the system was perceived and integrated within healthcare settings. Respondents were sampled because of their knowledge of VLN and their experience in using or evaluating the system.

### Ethics

We obtained ethical approval from the School of Social and Political Science at the University of Edinburgh. Participants were provided with a consent form and an information sheet describing the study aims, procedures, and data management practices before participating in the study. Participants were allowed at least 48 hours to consider whether they agreed to participate and provided written informed consent. The participants were informed that they were free to withdraw at any time and that their responses were not identifiable (ie, all personally identifiable information was removed from interview recordings and transcripts were anonymized).

### Sampling and recruitment

This study was conducted between February and December 2022 in 5 hospitals implementing VLN for screening incidental CT scans for lung nodules. We also interviewed patients with respiratory conditions and implementers including respondents from outside these hospital settings who could provide us with insights surrounding the implementation and adoption of AI in radiology. We liaised throughout the study with the project manager of the software company who provided contact details of the local hospital implementation leads. We began recruitment with these individuals and identified further participants by asking for recommendations of others who had an interest in or experience of AI-based radiology imaging. Our aim was to sample for maximum variation in terms of demographics, experience, and expertise.

Patients were recruited via gatekeepers at charities for patients with respiratory conditions, including Asthma and Lung UK and the Roy Castle Foundation in the United Kingdom. We included a wide range of patients from various demographics, although none had experience or knowledge of VLN. Wider stakeholders were sampled via our professional networks and included academics who had researched AI radiology imaging systems, as well as system developers of other AI systems in radiology. To attract radiologists, we offered to reimburse them for taking part in interviews.

### Data collection

The research team (KC, RW, NF, SH) had a background in qualitative research in healthcare and developed the interview guides in collaboration with a consultant radiologist from the study team (RR). The topic guides for clinicians, experts, implementers, and patients (Table 1) were amended in discussion with the research team as interviews progressed to include themes that emerged during interviews. Key lines of inquiry for all groups included their level of knowledge, understanding and experience of AI tools in healthcare. For clinicians, strategic decision-makers, suppliers, and academics, we were also interested in implementation and adoption experiences, perceived impact on care provision and organizational functioning, and any concerns and challenges experienced. Interviews were conducted by two researchers (NF and SH) via Microsoft Teams or in person. Interviews were audio-recorded and transcribed verbatim.

**Table 1.** Topic guide for clinicians and implementers, and patients.

Clinicians and implementers
**Section 1: General introduction**
1. Please can you tell me about your role in your organization and how you are involved with patient care? 2. In your day-to-day work, how often do you work with AI tools? 3. What kind of AI tools do you use? 4. Do you think your practice has changed in any way because of using AI tools? 5. Can you please provide some examples?
**Section 2: Knowledge and understanding of AI tools**
6. What are your general attitudes toward the use of AI in your work? 7. What are your patients’ understanding about the use of different AI tools and how they aid the diagnosis? (Including any benefits or drawbacks) 8. In your experience, how does AI help you in your everyday work?
**Section 3: Experience and opinions of AI**
9. In your experience, how does/can VLN impact on your care provision? (Prompt: can you give me some examples of and the effects on the patient population or your practice?) 10. Specifically, does VLN help you with making a diagnosis? If so, how? 11. Do you have any concerns about AI such as VLN? (eg, data storage, Information Technology (IT)/cyber security, management?) 12. Is there anything else you would like to share with me that you feel is relevant to the use of AI that was not covered during our discussion?

**Patients**

**Section 1: General introduction**
1. Please can you tell me about your chest condition and when were you first diagnosed? 2. How many chest scans have you had to date? 3. Do you know if your physician has used AI to scan your chest? 4. Do you understand the reports your physicians give you about your results, including AI results? 5. Do you ever ask any questions about AI tools your physician may be using to diagnose you?
**Section 2: Patient knowledge, understanding and attitudes toward AI**
1. What are your general attitudes toward the use of AI in healthcare? 2. What is your understanding of the use of different AI tools and how they aid the diagnosis? (Including any benefits or drawbacks) 3. In your experience, to what extent are you as a patient informed about the way AI helps physicians/clinicians make a diagnosis? 4. (Explain the tool if needed) Now that you know more about AI tools, would you specifically request to have a chest scan that would include AI tools? 5. Any other comments regarding AI and your chest condition?

### Data analysis

Transcripts were anonymized, numbered, and coded by two researchers (SH and NF) using NVivo (QSR International, v.12). We used thematic analysis to identify common patterns (themes) across transcripts.^32^ Codes were applied inductively (conceptualized prior to analysis), and deductively drawing on the Technology, People, Organizational and Macroenvironmental factors framework.^33^ We also held two analysis workshops, where we explored tensions and trade-offs offs in the data by presenting emerging findings to the wider research group. This resulted in minor modifications to the narrative, mainly relating to provision of additional detail in relation to clinical workflows and software functionality. Themes were checked by two researchers (KC and NF) in an iterative process to reach an agreement on the Results narrative.

## Results

We interviewed 39 people (see Table S1), with each interview lasting between 15 and 76 minutes. Twenty-two interviews were conducted with clinicians. These included 11 consultant radiologists, five project managers or specialists in clinical imaging information systems, three trainee radiologists, two radiographers, and one chief clinical information officer. 12 interviews involved patients with long-term chest conditions, such as lung cancers, asthma, and chronic obstructive pulmonary disease. All patients had undergone chest CT scans and received care for their conditions, some for over a decade. Five interviews included other experts in the field such as academic researchers with a background in radiology AI. We contacted all eight implementing sites, but 27 clinicians did not reply to the invitation for an interview. They were followed-up once.

Detailed participant characteristics are provided in Table S1. All except two clinicians had been using VLN regularly in their work as part of screening programs. During the time of data collection, sites had used VLN for varying lengths of time, ranging from six months to over five years. Accordingly, users reported varying levels of experience. In some clinics, all radiologists and radiographers used VLN, in some clinics both radiographers and radiologists used the tool, and in some, the interviewed clinician was the only user of the tool. 22 interviewees were male and 17 were female. Clinical users came from 10 different sites. Wider stakeholders were located in The Netherlands and in Belgium.

The findings are summarized in Table 2. We will discuss each theme and subtheme in the following paragraphs.

**Table 2.** Summary of main themes.

Themes	Subthemes
1. Perceived drivers and benefits	Anticipated and early experienced benefits Benefits vary with differing usage, skills, and workflows
2. Design of the tool and integration	Understanding and compensating for system limitations Integration with existing health information infrastructures delayed VLN rollout
3. Appropriation of the tool by expert labor	The evolving role of radiologists AI may help to upskill some staff
4. Clinical governance, quality assurance, maintenance, and post-market surveillance	Governance and professional scrutiny Post-market surveillance, ongoing quality assurance, and maintenance Sustainability and scale-up

The following paragraphs will describe each of the themes and subthemes in detail and provide supportive quotes from the data.

### Theme 1: Perceived drivers and benefits

#### Anticipated and early experienced benefits

Overall, VLN was seen to be usable, as it integrated directly with the PACS system and required little additional effort by users to view results. It was therefore readily adopted.

The good thing about VLN is that it’s incorporated into our PACS system or any program that we would have used anyway for reporting. In terms of the workflow, it either wasn’t disturbed, or it was very minimally disturbed for the radiologist, it’s not like you had to go to a different room or a different computer, you know, you might just have to change to a different screen. (Consultant radiologist, site B)

Participants reported that VLN made the process of interpreting images faster and provided details that were not easily perceived with the human eye (eg, nodule volume). This in turn impacted on clinician’s confidence in making a diagnosis.

I thought that it would make us quicker and more efficient and it has. Without a doubt, it’s very good at picking up little nodules that would be difficult to pick up with the naked eye, and, therefore, it does make the process much easier and quicker… (Consultant radiologist, site F)

Radiologists described using VLN as what they referred to as a “second reader,” increasing confidence in their clinical decisions over time and reducing anxiety that they may have missed a nodule. This confidence in the technology led to increased perceived efficiency, as radiologists could now focus their time and attention on complex cases:

It’s like having a second eye for the radiologist. We all miss things, we’re human beings, but having sort of a second pair of eyes, a computer program scanning the scan and picking up a nodule that you may potentially have missed is definitely an extra reassurance for us, but obviously better for patients as well. (Consultant cardio-thoracic radiologist, site D)

Time was a factor in the uptake and integration of the technology in everyday workflows. Clinicians with longer user experience with the tool were more confident and familiar with its use, indicating that it took time to learn about the performance of the tool, establish how it might be reliably used and integrated into their everyday workflows and practices. The initial period of familiarization was very short (no more than three months). VLN was designed for easy adoption by aligning with existing workflows. System maintenance was provided by the developer for each site. This also included a service for queries, and local configuration, including adjustment of sensitivity and specificity, which clinicians found valuable because it allowed them to set parameters for their specific patient population.

[…] anecdotally, people have said that it has improved efficiency, so as they’ve become more confident using the technology and realized that it is functioning very well. They will not necessarily do their usual extremely in-depth checks because they’ll know the system will do it […] there is a lot more detail provided by the software on the types of nodules, the size and volumes and changes over time, than we would have been able to do previously. (Consultant interventional radiologist, site C)

Radiologists found VLN to be superior to the earlier computer-aided detection (CAD) systems (eg, embedded in scanners), which were perceived to be cumbersome and consequently often not used. Importantly, VLN automatically calculated some information—such as nodule volume—that previously had to be manually measured and laboriously estimated. This resulted in faster and more accurate assessment of the size and growth of nodules. VLN also included tools for generating reports, which was seen as advantageous and time-saving.

So previously […], you would have had to put up a different icon on your desktop, type in your patient’s name or hospital number, find their study, open their study in the specific workflow package, and then click, run CAD [computer-aided detection], and then review the output. Obviously, what VLN does is - it mostly generates a labeled additional DICOM [Digital Imaging and Communications in Medicine] image at the point that you open up the study, right, and then it’s all there. So, when VLN nodule analysis is done, it massively cuts down the time […] And then when it is there, the volume is available. (Consultant radiologist, site A)

Implementers felt it would improve accuracy of detection and some suggested that the technology could help to reduce compensation claims for missed lung nodules (though there was no direct evidence for this).

I think from our [hospital], we are very much of the opinion that given the number of serious incidents that have occurred because of missed lung nodules and stuff, they would happily invest in the technology as a way to reduce that risk because paying out half a million pounds because of a missed nodule and the harm done to a patient eventually, by missing a nodule, having cancer and things, it’s considerably cheaper and more sensible to just pay for a product like this, that can help, even if it’s not 100% accurate. (Consultant radiologist, site F)

Patients we interviewed also anticipated benefits from AI technology. Their general attitudes were very positive, with most patients stating that they had understood the purpose of AI, and more so when the researchers explained it to them. Patients were generally positive about AI in the hands of radiologists because they trusted radiologists were using the technology appropriately. They also trusted healthcare organizations to procure systems appropriately. Several patients had mentioned they would choose a hospital where AI was used to inform clinical decisions if they had this knowledge and choice.

I, to a certain extent, yes, I do trust the consultant. And I’m sure he wouldn’t suggest it unless he thought it was something helpful for me. I mean, they wouldn’t waste the resource as the NHS is stretched to a breaking point. I don’t think they would be using that kind of diagnostic tools unless they felt it was something that would benefit the patient or contribute to research. (Female, 80-90, asthma and bronchiectasis, England; did not have an AI scan)

#### Benefits vary with differing usage, skills, and workflows

The benefits of VLN were seen to depend to some extent on the workflows and division of labor through which the tool was adopted. Having VLN functionality was perceived to help getting all the information required and potentially saved time and reduced unnecessary follow-ups.

Some radiologists don’t do volumes so, of course, they’ll just say, follow-up, when, actually, they probably don’t need a follow-up and then we end up discussing at an MDT [multi-disciplinary team] and then we’ll do a volume, then discharge them. There’s another extra step that is probably not needed. From that point of view […] it depends on the clinical confidence of the reporting body radiologist, really, but it could, potentially, save a lot of patients being referred into our service. (Radiographer, site D)

Radiologists within a cardio-thoracic specialty reported used VLN differently than general radiologists, to collect more detailed information, such as the dimensions of the nodules, instead of just detecting the presence of nodules.

Someone who’s not necessarily a cardiothoracic radiologist, may just say there is a nodule, follow the guidelines. Whereas a cardiothoracic radiologist is more likely to say, the nodule is there, and it’s this volume, the British Thoracic Society (BTS) guidelines advise you to do X, Y and Z, and allow you to give a bespoke follow-up suggestion. (Consultant cardio-thoracic radiologist, site D)

We also found differences in use varying with experience. For example, more experienced radiologists were more confident in making clinical decisions without VLN. They also felt better able to discount probably erroneous instances picked up by VLN and were concerned that less experienced users would rely on the machine’s judgments (which may in turn lead to unnecessary follow-up).

And I was thinking that for people who are less familiar with BTS guidelines and nodules, if it picks up multiple nodules that don’t necessarily need to be followed-up, but they’re less familiar with it, they might then put the patient into a follow-up program. And the patient is going to be recalled for further CT scans that they might not definitely need. (Consultant chest radiologist, site B)

### Theme 2: Design of the tool and integration

With use and experience, radiologists’ confidence in VLN grew. However, there was also an awareness that radiologists should not be entirely reliant on or overconfident in the results of AI. They reported getting used to double-checking the results of the system. This took up some time but much less time than scanning the image without the use of VLN.

So I’m confident that the system will pick up basically everything that looks like a nodule, that smells like a nodule, even if it’s not and where I think, yes I’m not really convinced about that, then I’ll look at the blind images but yes, it’s reduced that time, in that respect and I no longer do all the really in-depth checks that I would have done previously with manipulating the images to make nodules look more obvious on the system because I know they will pick them up, so I will just look at that and just go, “yes fine”. (Consultant interventional radiologist, site C)

#### Understanding and compensating for system limitations

Radiologists followed specific guidelines and internal audits of quality assessments using standard datasets on which they periodically assessed themselves. They were able to use the same procedures to assess the performance of the AI tool. Although the internal operation of VLN was not necessarily understood, its outputs were scrutinized in forensic detail. This in turn allowed users to rely selectively and appropriately on tool alerts.

Yeah, so I’ve reviewed the lungs…in my normal way, so I’d usually review both…and then go through the VLN tool on both as well. And then I would check the nodule software on the PACS system, to see if I’ve missed anything essentially, and then re-review the imaging and correlate those findings if it came up with something I hadn’t seen. (Consultant radiologist, site B)

Some radiologists had specifically recognized parts of the chest and parts of the image where the tool may not produce accurate readings. One example was an area in the lungs (in the central midline portion of the thoracic cavity) where, due to the presence of blood vessels, the AI may have “blind spots” and not produce precise results.

It doesn’t cope very well with identifying masses that are in the area of the lungs where the blood vessels interface with the head and the mediastinum. That’s an area where it can be a bit of a blind spot even for big lesions for the software. Then the other area that it struggles with sometimes is lesions that are in the airway itself, so central airways. I think knowing that means that a human will specifically review those areas very carefully to make sure there’s nothing in those areas because we know that’s an area of potential blind spot or weakness for the AI. (Consultant radiologist, site F)

Over time, users learned how to work around the limitations of the system, identifying which areas produced erroneous results and which parts were reliable. Where these factors were likely to produce false negatives or false positives, they were therefore equipped to dismiss these.

[…] sometimes the AI software would draw round a nodule, but it might also draw round something that wasn’t a nodule, like a vessel, or like a benign pleural plaque or something like that. And we would sort of call those false positives. But we would just ignore that, it didn’t sort of take up lots of our time. (Consultant radiologist, visiting professor, Belgium)

Although experienced clinicians felt able to make a critical assessment of the output of VLN, this did not extend to patients. However, patients with good rapport with their clinicians stated that they would trust a report if it was produced by AI. A few patients mentioned that being shown the report from VLN would be useful.

The first high-resolution CT scan I had; I saw the consultant not long after that. In fact, she was really lovely. I was with her for quite a few years. But she’s now moved on. So, I’m now with different consultants. And she was very good. She showed me the scan on the screen, and she explained what had been going on. (Female, 70-80, lung cancer, England; did not have an AI scan)

A few clinicians also reported that for patients who had many scans, VLN could only compare the current scan to the last (prior) scan. This in turn limited the ability to trace volume changes over an extended period.

[…] that ability to volume track, historically, over a range of scans, rather than just one scan, I think is something which would really lift the software to another level, and actually make it really useful. (Specialty registrar interventional radiologist, site J)

#### Integration with existing health information infrastructures delayed VLN rollout

VLN was designed to integrate with PACS to not interrupt the existing workflows of radiologists. We did, however, observe some implementation challenges relating to Information Governance and integration with local information systems (including PACS systems and electronic health records). This created teething problems such as delays with the planned rollout where the software developer was dependent on complementary product suppliers (eg, PACS, medical imaging cloud solutions, available IT engineers on-site) to resolve these issues.

There’s some communication issue between the […] server and our PACS so it’s…yes, it should have gone live probably about a month ago but nothing’s happened. (Radiographer, site E)

Similar problems arose in hospitals with limited internet connectivity. Interviewees reported time delays in VLN reporting in some hospitals due to weak local infrastructure setups, resulting in the tool being used asynchronously, which could disrupt workflows.

If I’m doing an in-patient scan and I’m on call, I aim to do reporting, near to live reporting, if possible. So, if the patient comes off the scanner, and I’m looking at it and VLN hasn’t had its chance to do its thing, I’m not going to sit about and wait for it. So, in this scenario, it hasn’t actually changed. And in that scenario, if I find a nodule, I’ll end up using the old workflow because ironically, in that scenario, until VLN is made quicker, you can’t use it. (Specialty registrar, site A)

Technical and imaging teams also noted that connection issues often delayed the implementation and full integration of the system into care provision.

It works automatically for 95% of the occasion… provided the worklists are up and running, […]. If we get an outage in which these things are done by 4G, so as with your mobile phone, dependent upon which site they go to, sometimes the signals are not as strong as in other sites. So, this is a bit of a weakness in this process. (Head of clinical imaging systems, site A)

There were also some concerns about cybersecurity and data storage requirements including associated costs.

People forget that storage does cost money. And if you’re suddenly processing, I don’t know, 50,000 studies [...] then it can potentially impact your storage. (IT project lead, site G)

### Theme 3: Appropriation of the tool by expert labor

#### The evolving role of radiologists

There was an overwhelming sense amongst interviewees that AI was changing care provision in radiology for the better. Although models for how VLN should be incorporated into the division of labor and workflows were still emerging, there was a general view that the role of radiologists would evolve positively with AI.^34^

AI won’t replace radiologists, but radiologists who use AI-enabled tools will replace radiologists who don’t. And that’s probably the way I see it from my standpoint, I see that AI is an incredibly valuable tool for radiologists to use. And that’s why I think we should be embracing these tools in our day-to-day practice. I think it’s anything that makes you safer, and secondarily faster, should be welcomed. (Consultant radiologist, site F)

However, some radiologists expressed concern about their role becoming undervalued with the evolving use of AI or changing public opinions perceiving them as “barely doing anything” (the notion also made famously by G.E. Hinton^35^). Nevertheless, there was also an insistence that radiology is a profession that requires a great deal of experience and skill.

In the past, when I was applying for radiology, like, five years ago now, the consultant that was helping me with my application said, “Oh, you definitely need to do interventional radiology, because that gives you practical skills and the AI can’t take that over”. But he says that, “otherwise, your job’s going to not be there”. I think I do worry that […] it would maybe degrade the opinion of the public or the people that pay us. (Specialty trainee registrar, site I)

#### AI may help to upskill some staff

The impact of the tool varied according to the skill of the user and their role in the division of labor. Although there was no sense that AI would replace radiologists, some mentioned that it may help to upskill some staff. There was a recognition from implementers, experts, and experienced clinicians that the tool output would make interpretation by a less experienced clinician easier and more precise. Here, the use of AI was seen to “democratize” imaging knowledge.

One of the key principles of using radiology AI is that it democratizes knowledge. So, you go from needing a highly pressurized expert in a very special part of radiology interpreting scans. You essentially have an AI assist, which means that people with less experience and less specialist knowledge can derive the same answers. So, my hope and expectation, I would say, is that anybody who [has good knowledge of] using PACS and a basic IT system can use, interact with, and gain benefit from using AI. (Consultant oncology radiologist, site G)

### Theme 4: Clinical governance, quality assurance, maintenance, and post-market surveillance

Governance, surveillance, quality assurance, and maintenance had a significant influence on adoption and procurement decisions. Participants were aware that actual performance in the field might vary as the tool moved from lab to field and from site to site. Radiologists were also aware of the lack of empirical evidence for AI-based applications in healthcare settings and had initial reservations about the system’s reliability.

I know there are shortcomings surrounding, obviously clinical utility, based on the lack of evidence and actually…when these algorithms work and they’re trained on the machine learning platforms with perfect 25-year-old chest x-rays and CTs in people that are completely normal but actually when you put it into the real world and you’re scanning 87-year-olds who are full to the brim of fluid and breathless, and does it still work, but actually do you see efficacy completely tail off and things? (Consultant radiologist, site B)

Some clinicians wondered how regulatory systems, such as the Medicines and Healthcare Products Regulatory Agency (MHRA), Care Quality Commission, NICE, the European Union Legal Framework, or the FDA would respond to the evolving nature of AI in healthcare settings in the future. Careful institutional and professional clinical governance by hospital organizations and staff with clinical responsibility for interpreting CTs complemented national regulation. Clinicians consistently emphasized that the responsibility for the final clinical decision lay with them.

I mean, you know it can only do relatively binary tasks at the moment, and those tasks are generally tasks that help radiologists, so I think there will be a way to go before it could report a whole CT scan, bespoke to the clinical information and the clinical referrer, and go through that sort of multi-faceted thought process. (Consultant cardio-thoracic radiologist, site D)

We further observed that in some instances organizations struggled to establish a business case for VLN. VLN was funded on a fee-per-scan basis, although sites were not being charged for scans during the trial. Organizations were unsure whether they would be able to develop a business case to justify continued use of VLN when free access to the technology supported by the trial ended.

Yeah, there’s funding for the first 12 months from [name of funder]. So, I’m unsure […] I think there was hope that it might continue to be funded. But I don’t know what the ongoing costs would be after, I think it’s September time. But that would be up to the clinical team to do their investment appraisal and everything else. So, they should be working on that now really. (IT Project Lead, former PACS manager, site G)

Senior managers noted that VLN competed with ongoing costs of other existing digital projects. They were aware of the high costs of procuring, validating, implementing, and optimizing stand-alone AI solutions focusing on one specific diagnostic application and looked for broader applications of AI in relation to lung cancers or lung diseases in general.

So, this is a really complex question to answer and, essentially, if we were to follow NICE guidelines, we basically have to show a health economic benefit within 12 months. And my feeling is that we may not demonstrate NICE’s gold standard of health economic benefits in 12 months, particularly given how expensive it is to get everything into one place. I suspect that maybe if we were to use longitudinal studies and observe this data for a bit longer, I suspect there will be financial benefits. (Consultant radiologist, site F)

#### Sustainability and scale-up

Failure to attend to environmental and organizational factors may impede acceptance and threaten the longer-term sustainability and scale-up of systems. These need to be considered during development and implementation. Clinicians, researchers, and implementers were aware of the potential technical and organizational challenges of scale-up across different sites. Software developers and local teams invested significant efforts in implementation. Technical teams (both within the hospital and outside) commented on their role as an intermediary between the software developer and hospital managers. Each site required bespoke configuration (eg, in relation to workflows). In some instances, there were also compatibility issues with installed PACS solutions, as described earlier. Many of these challenges became visible only post-implementation and implementation teams (and third-party suppliers) had not always made sufficient resources available for these activities.

It gets very technical, but what’s crucial is having something like [name of the company], in the middle of it all, connecting all the dots, because getting a single workflow is perhaps unlikely given the NHS and how each [hospital] operates, having that flexibility and the functionality to accommodate to, as we’re needed.[…] So, we facilitate the scan part of it where it’s outsourced to third parties. And then we also do the report component as well […] And that’s where, you know, our vendor [software developer] neutrality comes into it, we will and can communicate with all sorts of vendors and appliances. (Senior application specialist, site H)

In one site, a configuration problem led to impaired performance triggered by a system upgrade. This raised issues about the ongoing management of AI tool configuration. However, there was at that stage no sharing of information between hospitals about tool performance statistics (eg, number of false positives or false negatives) or implementation issues. This was partly due to information governance restrictions in implementing sites.

## Discussion

### Summary of findings

Our work showed that VLN was perceived as usable and useful by clinical users as a decision-support tool and as a “second reader.” There were some differences in use between expert and novice clinicians in that experienced radiologists rapidly became confident in using the tool in an efficient and reliable way, discounting probably erroneous instances, though noting that less experienced users might lack the skills and confidence to make these judgments. We also found a general view that the role of radiologists would evolve positively with AI and might facilitate re-skilling.

Based on the trust they had in their clinicians, patients also viewed VLN positively. The tool was designed to integrate within existing workflows and was readily adopted. Users became proficient over time as they learned the strengths and limitations of system performance. Detailed knowledge of the performance of the tool allowed them to rely selectively and appropriately on tool alerts, enabling responsible and dependable use.

Our work further highlighted contextual variations in tool performance and use between different hospital sites and different use cases and workflows depending on specialty and experience. We also showed how AI tools need to be integrated within complex existing infrastructures. This was not always easy (and integration with PACS systems was one of the key perceived issues associated with system usability). Providers highlighted the need to attend to ongoing quality assurance and maintenance.

Organizations were concerned that the initial and ongoing costs surrounding tool procurement, implementation, maintenance, and information governance might present challenges for establishing a business model of adoption and sustained use of these systems unless effective systems for handling these issues were established.

### Strengths and limitations

We explored the views of a wide range of stakeholders including specialist chest clinicians, patients, and other implementers working in radiology settings across the UK to gain high-level insights into the adoption and implementation of diagnostic AI in healthcare settings.

However, there are also some limitations. Firstly, some interview data were obtained from chest radiology specialists most of whom had experience of using VLN. A broader range of different types of users with various levels of experience with VLN may have provided more nuanced insights into different use cases (eg, between screening or routine care; general radiology or specialist lung cancer radiology centers). We also struggled to secure access to a large number and a wide range of organizational stakeholders as these were managing challenging workloads. Secondly, technical deployment issues in several hospitals participating in this study affected the progress of the trial and impacted on recruitment of participants. Nevertheless, we have provided an overview of issues that need to be considered when implementing, adopting, scaling, and maintaining diagnostic medical AI. Thirdly, the evaluation of VLN was largely focused on routine hospital practice where nodule detection is often incidental, but our respondents frequently drew on their experiences from lung cancer screening, where the focus is to identify lung nodules in an at-risk population. More detailed work is needed to characterize how the tool is integrated into different care pathways and shaped by different practices, workflow, divisions of labor and skills. Fourthly, it was difficult to gather the views of patients about VLN, as they had no direct experience with the technology. As a result, their views were relatively generic. The quantitative study of VLN implementation is still ongoing and this qualitative evaluation did not collect quantitative data about the performance of the tool. We also did not obtain any cost-effectiveness data, which would help to inform organizational procurement decisions. We did not know at the time of write-up if sites would keep using the system after the free trial had ended. These areas are the subject of ongoing work.

The sites in the study also received high levels of support from the software developer during the implementation of VLN, including the provision of training, integration with existing systems, and governance processes. Sites had extensive contact with a dedicated project manager, who logged and fed back their concerns. This extent of assistance offered by the software developer is unlikely to be sustainable in future implementations.

Lastly, collaboration with a software developer may be viewed as a potential conflict of interest. However, the research team remained independent throughout the study as an external evaluator. The software developer did not influence the views of the research team or the study findings.

### Integration of the findings with the current literature

Building on the literature surrounding complex health information infrastructures, there is no agreed method for successfully implementing diagnostic AI in radiology across different settings (ie, what may work in one setting may not work in another).^36^^,^^37^ Some of the emerging issues echo the relatively well-established evidence base in knowledge-based CDSS. For example, previous work has highlighted the importance of effective integration with workflows in order to minimize risks associated with alert fatigue. Mitigating factors have been found to include nonintrusive alert presentation and interface design.^38^^,^^39^ This is echoed in our work, where the integration with the PACS system meant that the interface was perceived to be nonintrusive and usable. Similarly, understanding and compensating for system limitations, as well as effective integration with existing health information infrastructures has been found to be a crucial factor in the implementation and adoption of CDSS.^9^^,^^40^

However, our work has shown that there are several distinct issues with AI-CDSS sustainability: (1) costs of stand-alone procurement and implementation of specific solutions; (2) scale-up and variations in performance across different sites with different demographic, technological, and organizational features; and (3) extension of the scope of AI solutions.

Almost all current advances in the field of AI fall under a narrow AI category, where AI is trained for one task only (eg, specific image recognition tasks, such as nodule detection on chest CT or hemorrhage on brain magnetic resonance imaging^5^). However, our work has shown that the contingencies surrounding point solutions may not fit within organizational business cases and procurement strategies, both in relation to implementation and ongoing maintenance.

We have shown how AI is currently being used responsibly and selectively by highly expert users, able to assess machine strengths and weaknesses. Less experienced users may tend to rely unduly on machine prompts.^41–43^ AI performance also needs to be subjected to ongoing scrutiny and there is a risk of degrading over time. As a result, even if an AI system works well in one organizational setting, this performance cannot be presumed to continue when use is extended to other organizations with different characteristics. Implementation of AI in a hospital setting is likely to involve changing workflow and clinical practices.^44^ Although these technologies may have become “domesticated” in some settings and workflows, this does not mean that they will easily be assimilated in others.^45^

Previous studies on diagnostic AI have not taken these contextual factors into account and have therefore not been able to consider an extension of the scale and scope of existing functionality.^46^ Our work suggests that this may, for example, involve exploring different use cases for more- and less-expert users of these systems (eg, as decision aids). Usage of these tools is liable to evolve. There is also ongoing discussion around the circumstances when AI is a decision-support tool, when it becomes a decision-making tool, and to what degree a human being needs to be kept “in the loop”.^1^^,^^47^^,^^48^ This will accentuate ongoing accountability concerns around who takes ultimate responsibility for patient safety issues: the clinician or the AI provider. We believe there is therefore a pressing need for more detailed studies of human-AI interaction.

### Implications for policy and practice

There are several recommendations emerging from our work. Most importantly, clinicians felt that they were ultimately responsible for clinical decision-making and used VLN as an assistive tool. We also learned that clinicians quickly came to understand the performance and shortcomings of the device and how to compensate for these. This reinforces work suggesting that we need to conceptualize AI-based systems in healthcare as assistive tools rather than autonomous decision-making entities.^49^ It also highlights the need to address (and educate users on) the strengths and limitations of systems in order for them to be able to develop ways to compensate for these.

In addition, we have shown that contextual factors impact the implementation and use of diagnostic AI-based tools. These, therefore, need to be considered throughout the design, procurement, implementation, and adoption process. There is, for example, a need to understand how AI-based tools may be included in existing care pathways (and related research on human-AI interaction and how this varies across different workflows and divisions of labor), how AI may be used to upskill a variety of stakeholders, and what unintended consequences such tools may have that may threaten their acceptance and sustainability.

The design principles and regulatory aspects of computer-based tools used in healthcare, including AI, are changing fast.^36^^,^^50^^,^^51^ Our study highlights the need for continued scrutiny of tool performance which may call for new post-market surveillance approaches. At this stage, however, we have little understanding of how this may be achieved or who might sustainably deliver it.

Finally, we identified three types of governance processes in this study: (1) risk governance by regulatory bodies such as the MHRA; (2) clinical governance by adopting hospitals; and (3) professional governance by the clinical experts involved. At this point, VLN is being deployed subject to detailed professional scrutiny, so the clinical user takes ultimate responsibility.^52^ The implementation and use of the tool are currently being conducted in a reflective, thoughtful, and responsible manner, but it is not clear that this level of scrutiny will be sustained as technology scales and extends in scope across medical fields and into different health service settings.

Although regulatory aspects of the work may only be transferable to a certain degree to other countries, as regulatory frameworks vary, the regulatory challenges posed by this technology are likely to be similar. The majority of our findings are therefore likely to be transferable to contexts outside the UK.

## Conclusion

Our findings highlight that VLN use is coevolving, as the tool is cautiously and responsibly exploited by skilled professionals learning how they may appropriately utilize AI strengths and compensate for its weaknesses. There is a need to develop clear models for how VLN should be incorporated into the division of labor and workflows in the future. In addition, our work has shown that despite high levels of clinical acceptability and usability, failure to attend to environmental and organizational requirements (including procurement costs) may threaten the longer-term sustainability and scale-up of the system.

## Supplementary Material

ocad191_Supplementary_Data

## Data Availability

The data underlying this article cannot be shared publicly due to the privacy protection requirements of patient healthcare data.
