# Supplementary material for: Early experiences of integrating an artificial intelligence-based diagnostic decision support system into radiology settings: a qualitative study
Source: J Am Med Inform Assoc. 2023 Sep 25;31(1):24–34. doi: 10.1093/jamia/ocad191 (PMC10746311; doi:10.1093/jamia/ocad191)
Supplement: ocad191_Supplementary_Data [file ocad191_supplementary_data.docx]

Appendix

**Participant characteristics**

| Number | **Other implementers** | Sex | Site |
| --- | --- | --- | --- |
| 1 | Academic respiratory physician | M | The Netherlands |
| 2 | Academic professor – medical image analysis, focus on pulmonary detection of CT scans | M | The Netherlands |
| 3 | Academic PhD student with interests in AI for radiology | F | The Netherlands |
| 4 | Academic professor – information and networks, socio-technical systems | M | The Netherlands |
| 5 | Radiologist, visiting professor | M | Belgium |
|  | **Healthcare professionals and specialist in clinical imaging systems** |  |  |
| 6 | Head of clinical imaging systems | M | A |
| 7 | Consultant radiologist | F | B |
| 8 | Consultant interventional radiologist | M | C |
| 9 | Specialty registrar | F | A |
| 10 | Trainee radiologist | M | D |
| 11 | Consultant cardio-thoracic radiologist | M | D |
| 12 | Radiographer | F | D |
| 13 | Radiographer | M | E |
| 14 | Consultant chest radiologist | M | B |
| 15 | Consultant radiologist | F | A |
| 16 | Consultant radiologist | M | F |
| 17 | Consultant oncology radiologist | M | G |
| 18 | Consultant cardio-thoracic radiologist | M | D |
| 19 | Consultant radiologist | M | F |
| 20 | IT project lead, previously PACS manager | F | G |
| 21 | Consultant radiologist | M | F |
| 22 | Chief clinical information officer | M | G |
| 23 | Chief operating officer | M | H |
| 24 | Consultant cardio-thoracic radiologist | F | I |
| 25 | Trainee radiologist | M | B |
| 26 | Specialty registrar interventional radiology | M | J |
| 27 | Senior application specialist | M | H |
|  | **Patients (Condition)** | Sex, Age | Site |
| 28 | Lung nodules (benign) | F, 70-80 | England |
| 29 | Stage 4 non-small cell lung cancer (NSCLC) | F, 70-80 | England |
| 30 | Lung cancer | F, 50-60 | England |
| 31 | Lung cancer | M, 40-50 | England |
| 32 | Bronchial neuroendocrine tumor | F, 40-50 | England |
| 33 | Lung cancer | F, 60-70 | England |
| 34 | Lung cancer | F, 60-70 | England |
| 35 | Lung cancer | F, 50-60 | England |
| 36 | Asthma, bronchiectasis | F, 40-50 | England |
| 37 | Asthma/lung condition | M, 50-60 | England |
| 38 | Asthma, chronic obstructive pulmonary disease | F, 60-70 | England |
| 39 | Asthma, bronchiectasis | F, 70-80 | England |
